# Identifying the Role of YAP in the Development of Rumen Epithelium Using 3D Organoid

**DOI:** 10.1155/sci/5105796

**Published:** 2025-07-11

**Authors:** Zebang Xu, Xinxin Xu, Yuling Mi, Yuanyuan Zhang, Qihua Hong, Bin Yang, Jiakun Wang

**Affiliations:** ^1^Institute of Dairy Science, College of Animal Sciences, Zhejiang University, Hangzhou 310058, Zhejiang, China; ^2^MoE Key Laboratory of Molecular Animal Nutrition, Zhejiang University, Hangzhou 310058, Zhejiang, China; ^3^Liangzhu Laboratory, Zhejiang University Medical Center, 1369 West Wenyi Road, Hangzhou 311121, Zhejiang, China; ^4^Department of Veterinary Medicine, College of Animal Sciences, Zhejiang University, Hangzhou 310058, Zhejiang, China; ^5^The Experimental Teaching Center, College of Animal Sciences, Zhejiang University, Hangzhou 310058, Zhejiang, China; ^6^School of Biological and Chemical Engineering, Zhejiang University of Science and Technology, Hangzhou 310023, Zhejiang, China

**Keywords:** hippo signaling pathway, organoid, rumen epithelium, YAP

## Abstract

Ruminants are of significant economic importance, and their unique digestive system features the rumen as a vital organ. The rumen is lined by stratified squamous epithelium, plays a crucial role in absorbing volatile fatty acids (VFAs) generated through microbial fermentation, thereby meeting the daily energy requirements of these animals. The maintenance of the rumen epithelium is a matter of concern. Here, we present compelling evidence that the hippo pathway effector yes-associated protein 1 (YAP) serves as a key regulator in maintaining rumen epithelial cells (RECs). Our findings indicate that rumen epithelial basal cells spontaneously undergo expansion and differentiation, ultimately forming organoids, and that the hippo signaling pathway is involved in regulating this process. Specifically, we demonstrate that YAP is indispensable for the initial specification and long-term maintenance of organoids. Activation of YAP promotes the growth and formation of these organoids, whereas inhibiting YAP hinders this developmental process. YAP activation exerts its effects by enhancing basal cells proliferation while simultaneously inhibiting differentiation. Conversely, YAP inhibition reduces the proliferation of basal cells. Notably, YAP activation promotes dedifferentiation of differentiated organoids. Moreover, YAP activation fosters intercellular tight junctions and strengthens cell-extracellular matrix interactions. In contrast, YAP inhibition reverses these features and leads to the disintegration of the organoids. Collectively, our data reveal the regulatory role of YAP in the rumen epithelium, which will help deepen the understanding of rumen development.

## 1. Introduction

Ruminants are significant economic assets, offering humans a high-quality source of protein [1]. The rumen is a unique digestive organ exclusive to ruminants, constituting 70% of the volume of the stomach. It digests forage and generates volatile fatty acids (VFAs), providing 70% of the daily energy requirements for the animals [2]. Therefore, the rumen serves as a vital target for nutritional regulation in ruminants. Promoting rumen development contributes to rapid early-life weight gain and enhanced growth performance, also serves as a means to meet the increasing global demand for animal-derived protein by the growing human population. Currently, some transcriptomic studies have reported that MAPK signaling pathway, Rap1 signaling pathway, Ras signaling pathway, Jak-STAT signaling pathway [3], hippo signaling pathway, Wnt signaling pathway, thyroid hormone signaling pathway, ECM–receptor interaction [4], PPAR [5], and IGF-1 [6] may be involved in the proliferation of rumen epithelial cells (RECs). However, there is still a lack of precise understanding of the mechanisms underlying rumen development, which constrains further precise nutritional regulation of rumen development.

Organoids are cultured products induced from stem cells in vitro, exhibiting similar cell types, and structural characteristics to those of native organs, and they represent a significant breakthrough in the field of cell biology over the past decade [7]. The development of organoids has provided valuable insights into the study of organ development, including that of the skin [8], esophagus [9], lung [10], stomach [11], intestine [12], and colon [13]. The rumen is an endodermal organ lined by stratified squamous epithelium consisting of the stratum basale, stratum spinosum, stratum granulosum, and stratum corneum [14]. Within the stratum basale, keratinocytes maintain the stratified epithelial structure through proliferation and differentiation [15]. We have recently established an effective protocol that allows isolated RECs to self-organize into organoids in vitro [16]. These rumen epithelial organoids exhibit tissue-like epithelial structure and cell types, and retain the capability for long-term expansion through serial passages.

The hippo signaling pathway has been identified as playing a role in regulating organ size and tissue regeneration [17]. YAP (yes-associated protein 1) is a transcriptional coactivator in the hippo kinase cascade, serving as a key regulator of developmental signals [18]. Upon entering the cell nucleus, YAP forms a complex with the DNA-binding TEA domain (TEAD) family transcription factors, initiating downstream transcription to regulate biological processes such as cell proliferation and differentiation [19]. YAP drives the expansion of stem cell and progenitor cell populations and plays a crucial role in the proliferation of various endoderm-derived tissues, including the esophagus, lung, and intestine [20–23].

In this study, we have discovered that the hippo signaling pathway may play a role in the development of RECs. Furthermore, we defined the role of YAP in the proliferation and differentiation of RECs. This will contribute to a better understanding of rumen development.

## 2. Methods

### 2.1. Ethics Approval and Consent to Participate

This study was approved by the Animal Care Committee of Zhejiang University (ZJU20230094).

### 2.2. Cells Isolation and Culture

Isolation of RECs and generation of rumen epithelial organoids were performed as described previously [16]. In brief, rumen epithelial tissue from five lambs at 5 days of age was collected postmortem and washed more than five times with phosphate-buffered saline (PBS; Biosharp, China) containing 10× penicillin/streptomycin (Solarbio, China) and 10 × gentamycin/amphotericin B (Yuanye Bio-Technology Co, China). The tissue was then cut into 5 mm sections and digested in 0.25% trypsin-EDTA solution (Solarbio, China) containing 10 μM Y-27632 (MCE, USA) at 37°C. The digestive solution was collected every 20 min, and RECs were obtained by centrifugation sat 300 × *g* for 5 min. The RECs were resuspended in cell culture medium (DMEM [Gibco, USA] supplemented with 10% [*v*/*v*] fetal bovine serum [CellMax, China], 5 μg/mL insulin [MCE, USA], 10 ng/mL EGF [MCE, USA], 10 μM Y-27632, and 10× penicillin/streptomycin) and incubated at 37°C and 5% CO_2_.

### 2.3. Organoid Culture

The RECs were resuspended in organoid culture medium (OCM, Table 1) at a density of 2 × 10^5^cells/mL, mixed with Growth Factor Reduced Matrigel (Corning, USA) on ice at a ratio of 1:4 (*v*/*v*) and placed in a 24-well plate as a 50 μL droplet, followed by incubation at 37°C for 30 min. Then 500 μL of OCM was added and the plate was incubated at 37°C and 5% CO_2_. Images were acquired daily using a microscope (TE2000-U, Nikon, Japan). The organoid formation rate was calculated as the ratio of the number of organoids to the number of seeded cells. Organoid diameter was measured using ImageJ (National Institutes of Health, USA).

### 2.4. Immunofluorescence Staining of Cells

Cells for immunofluorescence staining were cultured on coverslips (Biosharp, China), and all staining steps were performed in 24-well plates. Cells were fixed with 4% paraformaldehyde (Beyotime, China) for 15 min, permeabilized with 0.5% Triton X-100 (Sigma, USA) for 20 min, and blocked with goat serum for 1 h (Beyotime, China). Cells were incubated with the primary antibody at 4°C overnight, followed by incubation with the secondary antibody and 4′,6-diamidino-2-phenylindole (DAPI) at 37°C for 1 h. Coverslips were then overlaid on microscope slides (CITOTEST, China), and images were acquired using a fluorescence microscope (80i, Nikon, Japan). Fluorescence intensity was analyzed using ImageJ. The antibodies used for immunofluorescence staining are listed in Table 2.

### 2.5. Immunofluorescence Staining of Organoids and Tissue

Matrigel was dissolved, and organoids were collected after 1 h of incubation with cell recovery solution (Corning, USA) on ice. Tissue and organoids were fixed in 4% paraformaldehyde at 4°C overnight and dehydrated with 15% sucrose for 2 days and 30% sucrose for 3 days at 4°C. The samples were then embedded in OCT (SAKURA, USA), sectioned into 10 μm-thick sections using a cryostat (Thermo Fisher Scientific, NX50), and mounted onto adhesion slides (CITOTEST, China).

Sections were soaked in PBS for 15 min and transferred to sodium citrate antigen retrieval solution (Solarbio, China), then heated in a 99°C water bath for 10 min. After cooling to room temperature, the sections were washed with 0.2% Triton X-100 for 15 min and blocked with goat serum for 45 min at 37°C. After incubation with the primary antibody at 4°C overnight, sections were incubated with the secondary antibody and DAPI for 1 h at 37°C. Images were acquired using a fluorescence microscope (80i) after mounting, and fluorescence intensity was analyzed using ImageJ.

### 2.6. Cell Counting Kit-8 (CCK-8) Assay

The RECs were seeded in 96-well plates at a density of 3 × 10^3^ cells per well overnight and then treated with GA-017 and Verteporfin. After adding 10% of CCK-8 solution (MCE, USA) and incubating for another 1 h, cell viability was assessed at 4, 8, 12, 24, and 48 h using a Spark multimode microplate reader (TECAN, USA) at a wavelength at 450 nm.

### 2.7. RNA Sequencing Experimental Design and Sample Preparation

A total of five biological replicates were used for the RNA sequencing, with RECs-induced organoids from each lamb defined as one biological replicate. For sample collection, organoids from 12 wells were pooled into one biological replicate on 6 h and day 1; organoids from eight wells were pooled into one biological replicate on day 3 and 5; organoids from four wells were pooled into one biological replicate on day 7 and 9. For sample preparation, Matrigel was dissolved following a 1-h incubation with cell recovery solution on ice. Organoids were collected by centrifugation at 300 × *g* for 5 min, resuspended in TRIzol (Aidlab, China), promptly transferred into 2 mL polypropylene cryogenic vials (Corning, USA), and swiftly immersed in liquid nitrogen for subsequent RNA extraction.

### 2.8. RNA Sequencing and Transcriptome Analysis

Total RNA was extracted using the total RNA extraction kit (Aidlab, China), and RNA quality was assessed using an Agilent 2100 bioanalyzer (Agilent Technologies, CA, USA). RNA sequencing was performed on the Illumina platform by Novogene (Beijing, China). Clean reads were aligned to the sheep genome (http://www.ensembl.org/index.html, Oar_rambouillet_v1.0) using Salmon v.1.5.1 [24], and transcript abundance was quantified as transcripts per million (TPM) for each biological sample. Differential gene expression analysis was performed using DESeq2 v.1.36.0 [25]. Time-course analysis was performed using Mfuzz v.2.60.0 [26]. Kyoto Encyclopedia of Genes and Genomes (KEGG) enrichment, Gene Ontology (GO) annotation, and Gene Set Enrichment Analysis (GSEA) were performed using clusterProfiler v.4.8.0 [27].Visualization of the results was carried out using ggplot2 v.3.4.2 [28].

### 2.9. Quantitative Real-time Polymerase Chain Reaction (qRT-PCR)

The cDNA was synthesized using HiScript II Q RT SuperMix (Vazyme, China). All amplification reactions were performed using the ABI7500 Real-Time PCR System (Applied Biosystems, USA) with the ChamQ universal SYBR qPCR Master Mix (Vazyme, China). All experiments were conducted in triplicate. Transcript levels of all genes were normalized to GAPDH using 2^−ΔΔCT^ method. The primer sequences are listed in Table 3.

### 2.10. Measurement of Permeability of 2D Organoids

Generation of 2D organoids was performed as described previously [16]. After 7 days of culture, the 2D organoids were apically treated with 5 μM Verteporfin. On day 0, 1, and 2, 0.5 mg/mL 4 kDa FITC-dextran (Sigma, USA) was added to the upper chamber. After 1 h of incubation, the culture medium from the basal chamber was collected, and fluorescence was measured using a Spark multimode microplate reader (TECAN, USA) at 490 nm excitation and 530 nm emission.

### 2.11. Statistical Analysis

Organoids from each lamb were analyzed separately. All data were subjected to multiple comparisons using Tukey's HSD test or Student's *t*-test with IBM SPSS statistics 25 software (IBM Corp., NY, USA) and visualized by GraphPad Prism 8.3.0 (GraphPad Software, USA). Data are presented as mean ± standard deviation, *p*-values of 0.05 or less were considered statistically significant.

## 3. Results

### 3.1. Morphological Changes During Organoid Growth

**Figure 1:**
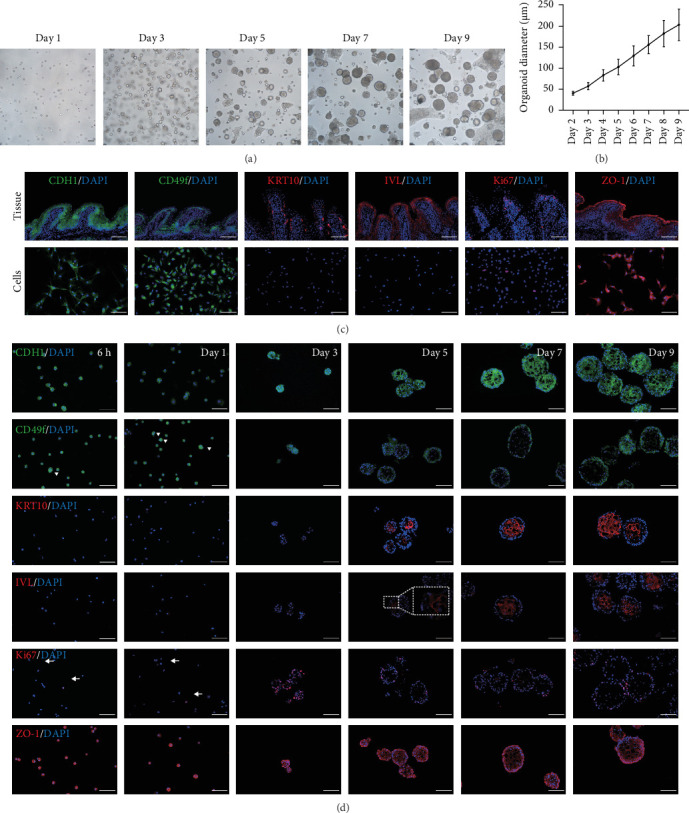
Morphological changes during organoid growth. (A) Representative images showing organoid growth. (B) Diameter variation of organoids. (C) Representative fluorescence images of rumen epithelial tissue and isolated rumen epithelial cells. (D) Representative fluorescence images of organoids at different culture times. Scale bars = 100 μm.

After 9 days of culture, single RECs formed rumen epithelial organoids with a diameter exceeding 200 μm (Figure 1A,B). Immunofluorescence images of the rumen epithelium and RECs are shown in Figure 1C. In rumen tissue, the epithelial layer exhibited positive immunofluorescence staining for CDH1 and ZO-1. Cells in the stratum basale were labeled with CD49f, while cells in the suprabasal layer were labeled with KRT10 and IVL. Proliferative Ki67^+^ cells were distributed within the stratum basale (Figure 1C). RECs used for organoid induction exhibited positive immunofluorescent staining for CDH1, CD49f and ZO-1, consistent with the staining pattern of cells in the stratum basale of the rumen epithelium (Figure 1C). Immunofluorescence images of organoids cultured for 6 h, 1, 3, 5, 7, and 9 days are shown in Figure 1D. All cells in organoids consistently exhibited CDH1^+^ and ZO-1^+^ throughout the culture process. During the first 3 days, CD49f^+^ cells rapidly expanded and became distributed in the outermost layer of the organoid as it grew (Figure 1D). KRT10^+^ cells appeared inside the organoids on day 5, and IVL ^+^ cells appeared on d 7 (Figure 1D).

### 3.2. Transcriptional Profile Dynamics During Organoid Culture

**Figure 2:**
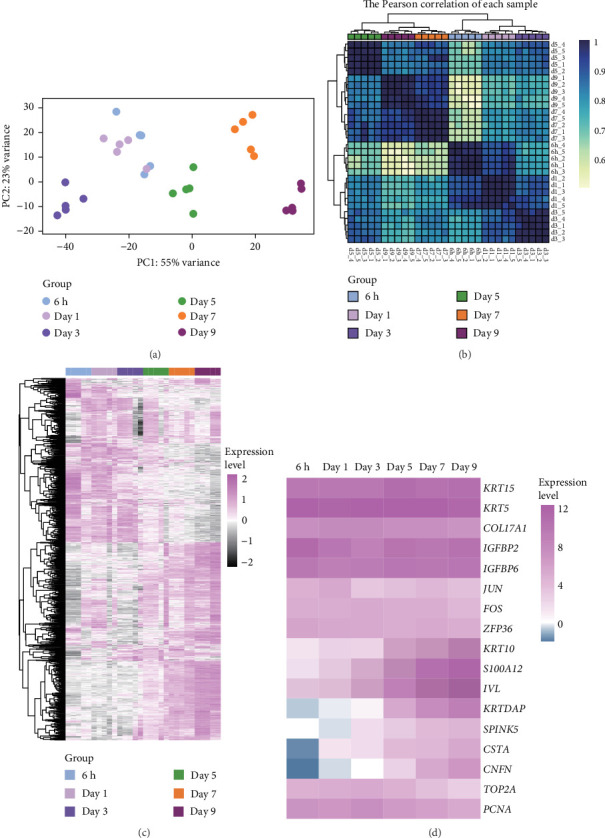
Gene expression profiles of the organoids at different time points. (A) Principle component analysis. (B) The Person correlation of each sample. (C) Heatmap showing the gene expression levels. (D) Heatmap showing the expression levels of rumen epithelial cell lineage-specific genes.

We next performed RNA-seq analysis to compare the gene expression profiles of organoids at different time points. Principal component analysis (PCA) was used to assess the expression profiles among all 30 individual samples (Figure 2A). PCA analysis clustered the samples according to different time points, confirming that the gene expression profiles were similar in organoids from the same time with minimal variation. The gene expression profiles of organoids changed dynamically over the course of culture, and organoids derived from different individual animals exhibited similar developmental patterns (Figure 2B,C). To investigate changes in cell composition during organoid development, we compared the expression level of cell-specific genes in organoids based on a published single-cell sequencing data set of sheep rumen epithelium (Figure 2D) [29]. Marker genes of basal cells, keratinocytes, and proliferative cells including *KRT15*, *KRT5*, *COL17A1*, *IGFBP2*, *IGFBP6*, *JUN*, *FOS*, *ZFP36*, *TOP2A*, and *PCNA* showed relatively stable expression levels throughout the culture period. In contrast, the expression levels of marker genes of differentiated suprabasal cells including *KRT10*, *S100A12*, *IVL*, *KRTDAP*, *SPINK5*, *CSTA*, and *CNFN* gradually increased with culture process.

**Figure 3:**
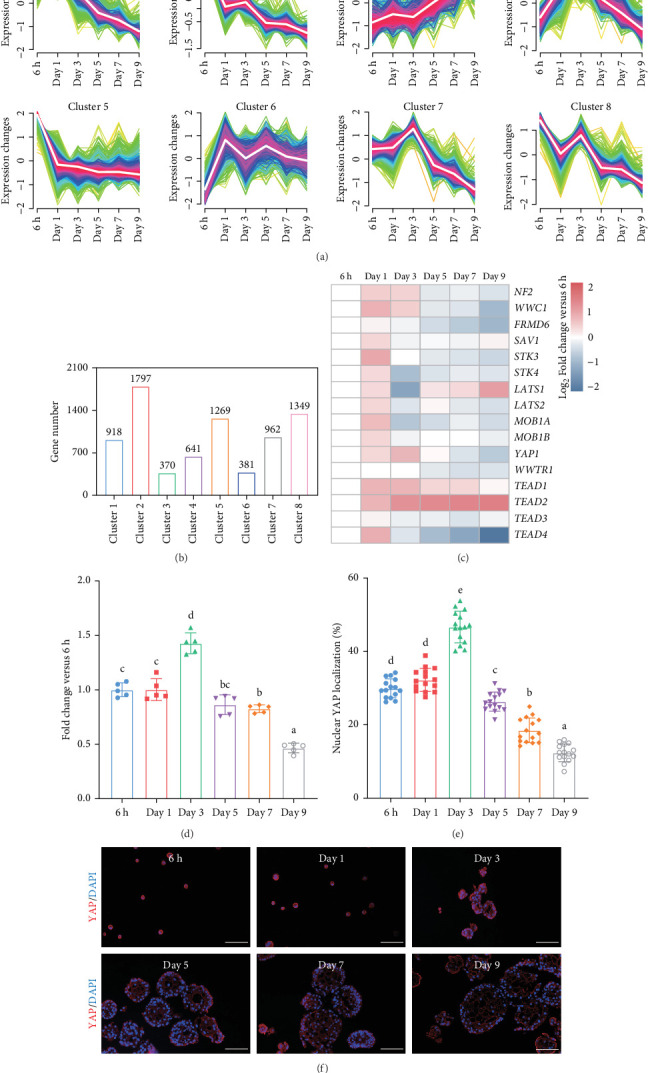
Time-series analysis. (A) Variation in gene expression levels across clusters. (B) The number of genes in each cluster. (C) Heatmap showing the expression levels of Hippo signaling pathway-related genes. (D) The relative expression level of *YAP* at different time points, *n* = 5 independent experiments. (E) Nuclear YAP localization ratio of organoids at different time points. Experiments were repeated for five slices per timepoint, three fields were randomly selected from each section to measure the fluorescence intensity. (F) Representative images of YAP staining at different time points. Scale bars = 100 μm. Different superscript letters indicate significant differences (*p* < 0.05).

We investigated temporal patterns of gene expression changes during organoid development using time-series analysis. A total of 8 clusters were identified (Figure 3A), and the number of genes in each cluster is shown in Figure 3B. KEGG enrichment analysis was subsequently performed foreach cluster (Figure S1). Notably, the hippo signaling pathway was enriched in cluster 1 and 4, which consisted of genes that exhibited high expression levels during the early stages of organoid development and decreased expression in later stages. Correspondingly, the expression levels of genes associated with the hippo signaling pathway showed a progressive decline as organoids matured (Figure 3C). The relative expression level of *YAP* mRNA displayed a dynamic pattern, initially increasing and reaching a peak on day 3, followed by a gradual decrease (Figure 3D). To further examine YAP activity, we performed immunofluorescence staining of organoids at different developmental stages. We observed a higher proportion of nuclear-localized YAP during the early stages of organoid development (Figure 3E,F).

### 3.3. Effects of Activation and Inhibition of YAP on RECs and Organoids

Considering that the hippo signaling pathway and its effector molecule YAP are involved in regulating numerous biological processes, including cell growth and organ development, we used GA-017 [30] and Verteporfin [31] to activate and inhibit YAP, respectively, and initially tested the effects of these two pharmacological agents on the growth of RECs.

**Figure 4:**
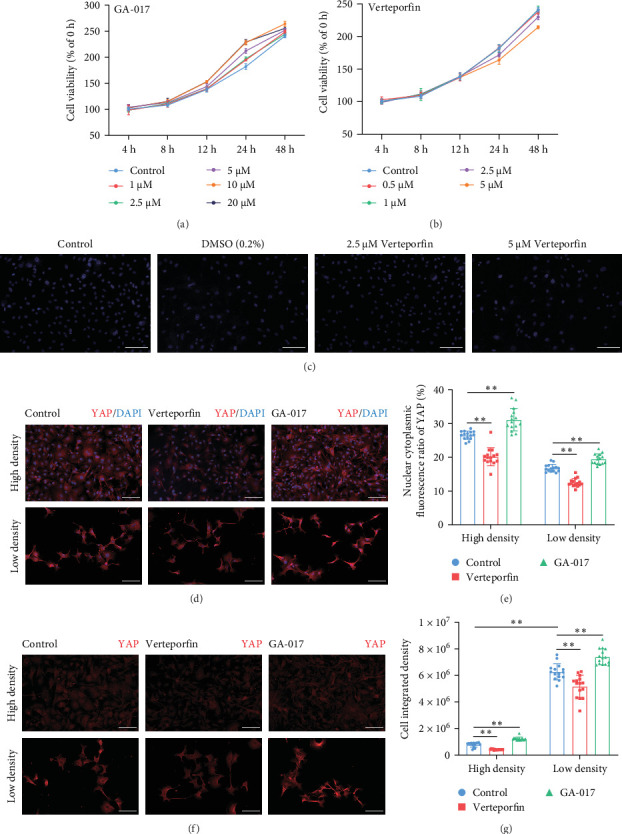
Effects of GA-017 and Verteporfin treatment on the proliferation of RECs. (A) Cell viability of RECs after GA-017 treatment. (B) Cell viability of RECs after Verteporfin treatment. (C) Hoechst staining of live cells after Verteporfin treatment. (D) Representative images of YAP staining of RECs treated with GA-017 and Verteporfin at high and low densities. (E) Nuclear-to-cytoplasmic fluorescence ratio of YAP. (F) Representative images of YAP staining under different cell densities. (G) Fluorescence intensity of YAP staining at different cell densities. For cell viability assessment, five independent experiments were performed and each experiment contains five wells of cells. For fluorescence intensity analysis, *n* = 5 independent slices, and three fields were randomly selected from each section to measure the fluorescence intensity. Scale bars = 100 μm. The *⁣*^*∗∗*^ indicates significant differences (*p* < 0.01).

GA-017 or Verteporfin treatment promoted or inhibited the proliferation of RECs, respectively (Figure 4A,B). With increasing concentration of GA-017, the relative viability of RECs gradually increased and peaked at 10 μM. As the concentration of Verteporfin increased, the relative viability of RECs gradually decreased. Treatment of RECs with Verteporfin for 48 h did not cause cell apoptosis, the results of Hoechst staining of live cells are shown in Figure 4C. GA-017 treatment significantly promoted YAP nuclear translocation, while Verteporfin significantly inhibited YAP nuclear translocation at both high and low cell densities. At high cell density, the average YAP fluorescence intensity per REC was significantly lower than that at low density (Figure 4D–G).

**Figure 5:**
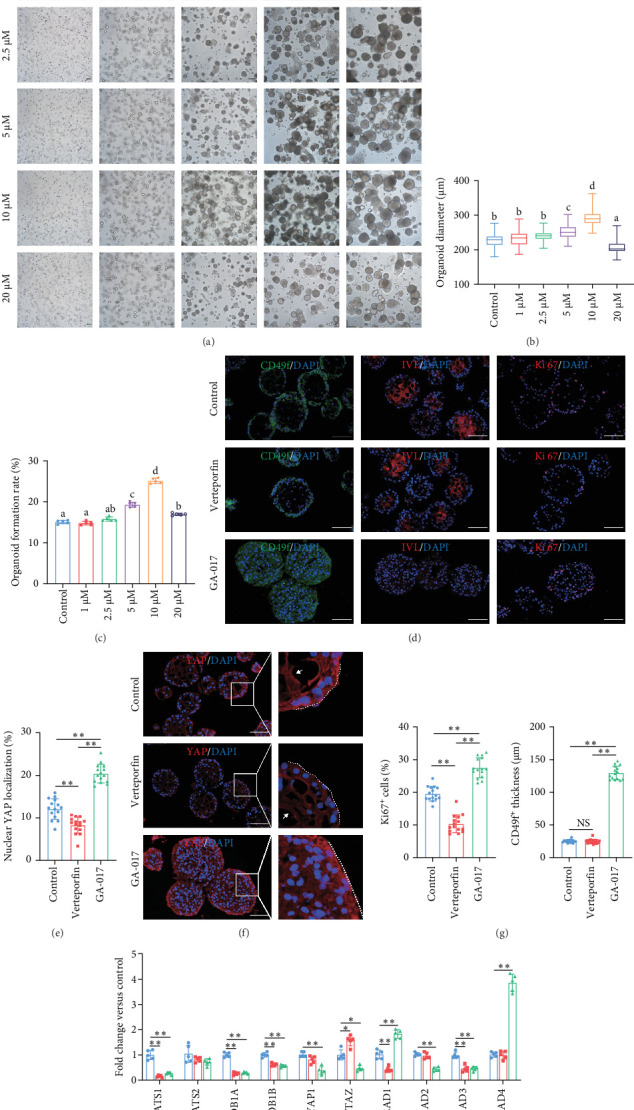
Effects of GA-017 and Verteporfin treatment on the organoid. (A). Representative images of organoids treated with different concentration of GA-017. (B). Diameter of organoids treated with different concentration of GA-017 on day 9, *n* = 5 independent experiments. (C). Formation rates of organoids treated with different concentration of GA-017, *n* = 5 independent experiments. (D) Representative fluorescence images of organoids treated with GA-017 and Verteporfin. (E). Nuclear YAP localization ratio of organoids treated with GA-017 and Verteporfin. (F). Representative images of YAP staining of organoids treated with GA-017 and Verteporfin. (G) Ki67^+^ cell proportion and CD49f^+^ cell layer thickness of organoids treated with GA-017 and Verteporfin. (H) The relative expression levels of Hippo signaling pathway-related genes of organoids treated with GA-017 and Verteporfin, *n* = 5 independent experiments. For fluorescence intensity analysis, *n* = 5 independent slices, and three fields were randomly selected from each section to measure the fluorescence intensity. Scale bars = 100 μm. Different superscript letters indicate significant differences (*p* < 0.05). The following were considered to indicate statistical significance: (*⁣*^*∗*^) *p* < 0.05, (*⁣*^*∗∗*^) *p* < 0.01.

Brightfield images of organoids treated with different concentrations of GA-017 are shown in Figure 5A. GA-017 promoted the growth and formation of organoids in a concentration-dependent manner. Organoids treated with 10 μM GA-017 exhibited the largest size and highest formation rate, while 20 μM GA-017 treatment inhibited the growth of organoids (Figure 5B,C). The normal function of YAP is necessary for organoid generation from RECs. RECs treated with low concentrations of Verteporfin lost the ability to assemble into organoids (Figure S2A). Mature organoids remained intact for 24 h after treated with 2.5 μM and 5 μM Verteporfin but began to disintegrate after 48 h (Figure S2B). Therefore, in subsequent trails, organoids were treated with 5 μM Verteporfin for 24 h. In GA-017-treated organoids, CD49f^+^ basal cells proliferated abnormally, the cell differentiation marker IVL was absent, and the proportion of Ki67^+^ proliferative cells was significantly increased (Figure 5D,G). In contrast, the proportion of Ki67^+^ proliferative cells were significantly reduced in Verteporfin-treated organoids (Figure 5G). GA-017 treatment significantly increased the proportion of nuclear-localized YAP in organoids, whereas Verteporfin treatment led to the opposite effect (Figure 5E,F). We determined the relative expression of hippo pathway-related genes in organoids by qRT-PCR (Figure 5H). In GA-017-treated organoids, compared with the control group, the expression levels of *LATS1*, *MOB1A*, *MOB1B*, *YAP1*, *TAZ*, *TEAD2*, and *TEAD3* were significantly decreased, while the expression levels of *TEAD1* and *TEAD4* were significantly increased. In Verteporfin-treated organoids, compared with the control group, the expression levels of *LATS1*, *MOB1A*, *MOB1B*, *TEAD1*, and *TEAD3* were significantly decreased, while the expression level of *TAZ* was significantly increased.

### 3.4. Transcriptional Profile Analysis of YAP-Activated and YAP-Inhibited Organoids

**Figure 6:**
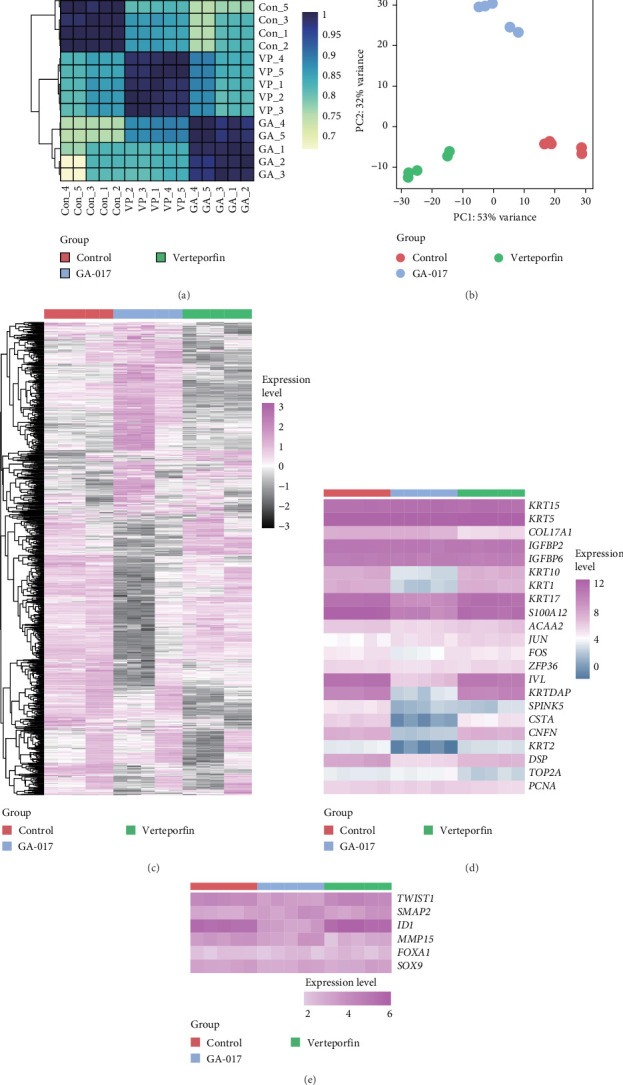
Gene expression profiles of the organoids after GA-017 and Verteporfin treatment. (A) The Person correlation of each sample. (B) Principle component analysis. (C) Heatmap showing the gene expression levels. (D) Heatmap showing the expression levels of rumen epithelial cell lineage-specific genes. (E) Heatmap showing the expression levels of cell EMT-related genes.

We compared the transcriptional profiles of organoids treated with 10 μM GA-017 and 5 μM Verteporfin. Organoids derived from different individuals exhibited similar transcriptional profile alterations in response to each treatment, while distinct transcriptional profiles were observed between the treatment groups (Figure 6A–C). The expression of marker genes for differentiated suprabasal cells (*KRT10*, *KRT1*, *KRTDAP*, *SPINK5*, *CSTA*, *CNFN*, and *KRT2*) was absent in GA-017-treated organoids (Figure 6D). GA-017 and Verteporfin treatment did not change the expression levels of genes related to epithelial-mesenchymal transition (EMT) in organoids (Figure 6E).

By comparing the transcription profiles of the GA-017-treated and the Verteporfin-treated groups with the control group, we identified 774 upregulated genes and 738 downregulated genes following GA-017 treatment, 290 upregulated genes and 1321 downregulated genes following Verteporfin treatment (Figure S3A,B). KEGG enrichment analysis was performed for the treatment group and the control group, and the top 15 pathways were selected based on fold enrichment (Figure S3C-F). GA-017 treatment upregulated pathways related to cell proliferation, differentiation, and death, including the hippo signaling pathway, TGF-β signaling pathway, and MAPK signaling pathway; pathways related to cell-extracellular matrix interactions, such as ECM-receptor interaction and focal adhesion; and cell junction-related pathways, including tight junction and adherens junction. GA-017 treatment downregulated pathways related to development, including signaling pathways regulating pluripotency of stem cells, Rap1 signaling pathway, Wnt signaling pathway, and axon guidance. Verteporfin treatment upregulated pathways related to cell growth and death, including mitophagy, NF-kappa B signaling pathway, hippo signaling pathway, TNF signaling pathway, mTOR signaling pathway, and MAPK signaling pathway, downregulated pathways related to cell-extracellular matrix interactions, including ECM-receptor interaction and focal adhesion; pathways related to development, including axon guidance and notch signaling pathway; pathways related to glycan biosynthesis and metabolism, including glycosaminoglycan biosynthesis-heparan sulfate/heparin, mucin type O-glycan biosynthesis, and N-Glycan biosynthesis.

To further characterize the dynamic trends of specific gene sets in organoids after treatment with GA-017 and Verteporfin, we performed GSEA, followed by KEGG enrichment and GO annotation. GA-017 treatment upregulated 15 KEGG pathways and downregulated 4 KEGG pathway, upregulated 182 GO terms and downregulated 74 GO terms (Figure S4A,C). GA-017 treatment upregulated KEGG pathways related to cell junction, including focal adhesion, tight junction, and adherens junction, downregulated signaling pathway regulating pluripotency of stem cells (Figure S4B). GA-017 treatment upregulated GO terms related to cytokine (cytokine-mediated signaling pathway, cellular response to cytokine stimulus, cytokine receptor binding, response to cytokine, and cytokine activity) and cell proliferation (positive regulation of cell population proliferation, chromosome segregation, DNA replication, nuclear division, and cell cycle) (Figure S4D), while downregulated GO terms related to transmembrane transporter (transmembrane transporter activity, monoatomic ion transport, transmembrane transporter, transporter activity, and channel activity) and epithelial differentiation (cell fate commitment, epithelium development, anatomical structure morphogenesis, epidermal cell differentiation, and keratinocyte differentiation) (Figure S4E).

Verteporfin treatment upregulated 19 KEGG pathways and downregulated 34 KEGG pathways, upregulated 285 GO terms and downregulated 456 GO terms (Figure S5A,C). Verteporfin treatment downregulated KEGG pathways related to cell junction (adherens junction, cell adhesion molecules, and focal adhesion) and development (signaling pathway regulating pluripotency of stem cells, Calcium signaling pathway, notch signaling pathway, Wnt signaling pathway, and axon guidance) (Figure S5B). Verteporfin treatment upregulated GO terms related to protein biosynthetic (Figure S5D), while downregulated GO terms related to development (regulation of multicellular organismal development, regulation of anatomical structure morphogenesis, multicellular organism development, cell morphogenesis, axon development, axon guidance, and axonogenesis) and cell junction (cell adhesion mediated by integrin, cell-matrix adhesion, cell junction assembly, cell–cell adhesion, and cell adhesion) (Figure S5E).

### 3.5. Effect of YAP on Cell Differentiation and Cell Junction of Organoids

**Figure 7:**
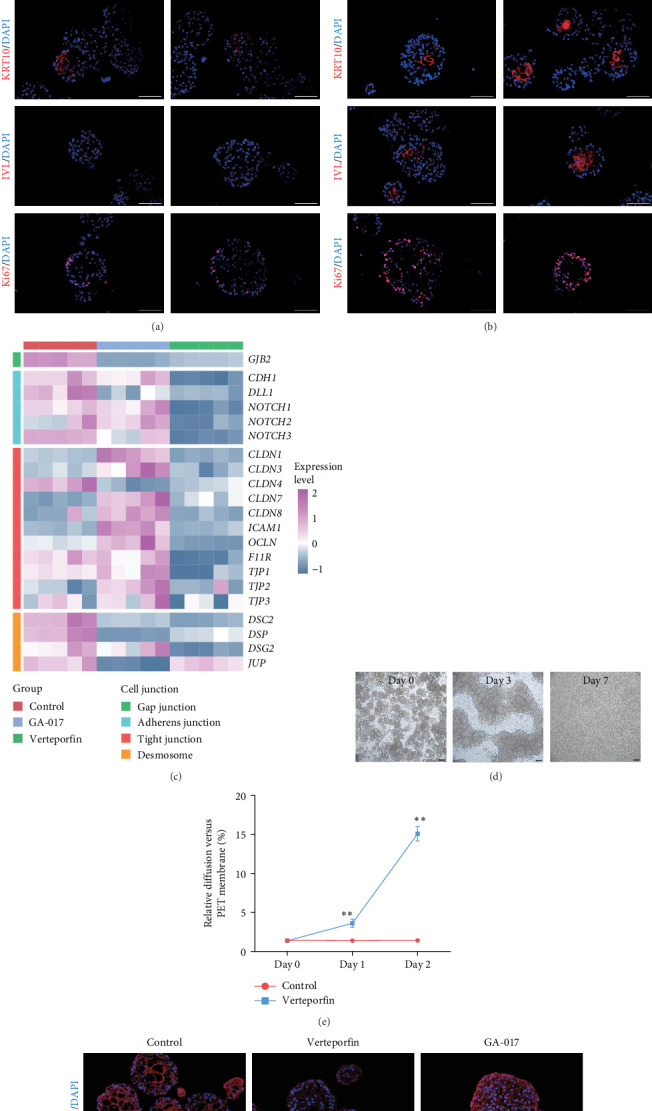
Effect of YAP on cell differentiation and junction. (A) Representative fluorescence images of organoids treated with GA-017 for 7 days, then removed GA-017 and cultured for another 3 days. (B) Representative fluorescence images of differentiated organoids treated with GA-017 for 3 days. (C) Heatmap showing the expression levels of cell junction-related genes. (D) Representative images of 2D organoids. (E) Permeability of FITC-dextran in 2D organoids after 0 day, 1 day, and 2 days of treatment with Verteporfin, normalized with cell culture inserts without organoids, *n* = 5 independent experiments, the *⁣*^*∗∗*^ indicates significant differences (*p* < 0.01). (F) Representative images of ZO-1 staining of organoids treated with GA-017 and Verteporfin. Scale bars = 100 μm.

To validate the transcriptome analysis results, we further investigated the role of YAP in organoid cell differentiation and junction. Organoids were treated with 10 μM GA-017 either during the first 7 days or the last 3 days of culture. Immunofluorescence staining showed that organoids began to differentiate after removing GA-017, KRT10 was detected in a small number of organoids, while IVL was absent in all organoids, Ki67^+^ proliferative cells were localized to the outermost layer of the organoids (Figure 7A). When differentiated organoids were subsequently treated with GA-017 for 3 days, they gradually lost their differentiation characteristics. CD49f^+^ cells formed a multilayered structure, KRT10^+^ cells were detected in a small number of organoids, and only a very small number of organoids still retained IVL staining. Ki67^+^ proliferative cells were no longer confined to the outer layer (Figure 7B).

Verteporfin treatment reduced the expression levels of genes associated with gap junctions, adherens junctions, tight junctions and desmosomes. In contrast, GA-017 treatment increased the expression levels of genes related to tight junctions but decreased the expression levels of genes related to desmosomes (Figure 7C). To further assess the effect of Verteporfin on epithelial barrier integrity, we generated 2D organoids on cell culture inserts (Figure 7D). Verteporfin treatment significantly increased FITC-dextran penetration (Figure 7E), indicating that intercellular junctions in 2D organoids were disrupted. Immunofluorescence staining of ZO-1 also confirmed the adverse effect of Verteporfin on tight junctions (Figure 7F).

## 4. Discussion

Ruminants possess the remarkable ability to convert low-value feed into high-quality animal-derived protein, making their primary energy producing organ, the rumen, a critical target for research in ruminant nutrition. The hippo signaling pathway is highly conserved in mammals and plays a central role in regulating organ homeostasis [32]. Its pivotal involvement in ruminant pregnancy recognition, embryonic development, and cystic ovarian disease has been well-established [33–35]. In recent years, stem cell-derived organoids, which retain the structural and functional characteristics of their origin organs or tissues, have become reliable models for in vitro experiments. Here, we utilized rumen epithelial organoids as a platform to demonstrate that YAP serves as a crucial regulatory factor in the proliferation of rumen basal cells. Furthermore, YAP may indirectly impact the development and maintenance of stratified squamous epithelium.

The in vitro assembly of isolated stem cells into organoids involves a sequential process encompassing both stem cell population expansion and differentiation. During the cultivation of hepatic organoids, the removal of exogenous Wnt signals promotes the transition of organoids from a progenitor cell phenotype to a mature liver cell phenotype [36]. Conversely, during the growth of esophageal and skin organoids, stem cell differentiation appears to occur spontaneously [20, 37]. This observation aligns with our findings: the proliferation and differentiation of rumen epithelial basal cells do not depend on external culture condition adjustments, with the hippo signaling pathway likely playing a predominant role. Throughout the cultivation of rumen epithelial organoids, genes associated with the hippo signaling pathway exhibit high expression levels during the basal cell proliferation stage and gradually decrease in expression during differentiation. In the skin, the daughter cells of keratinocytes lose YAP nuclear localization and initiate differentiation after losing contact with the basement membrane extracellular matrix [38]. This may potentially explain the spontaneous proliferation and differentiation of rumen epithelial basal cells, as the contact between cells in organoids and the extracellular matrix is absent. Surprisingly, we found that activating YAP upregulates signaling pathways related to cell-extracellular matrix interactions within the organoids, while inhibiting YAP yields the opposite results. This suggests a bidirectional relationship between YAP and cell-extracellular matrix interactions.

We have observed that within rumen epithelial organoids, YAP is retained within the nuclei of basal cells, while it is sequestered in the cytoplasm of differentiated cells. This discovery aligns with previous findings in lung, esophageal, and epidermal basal cells [20, 38, 39]. In the case of esophageal organoids, the application of Verteporfin, a YAP inhibitor, led to a reduction in the proliferation of esophageal progenitor cells, resulting in a decreased proportion of Ki67^+^ proliferative cells [20]. In the context of mouse skin, the number of Ki67^+^ cells significantly reduced after YAP knockout [38], which is consistent with our observations. Significantly, we have identified that the inhibition of YAP impedes the early expansion of basal cells during the formation of rumen epithelial organoids and hinders the long-term maintenance of mature organoids in vitro. This highlights the critical role of YAP in governing the early development and sustained maintenance of rumen epithelial basal cells. In epidermal and tracheal epithelium, the cytoplasmic localization of YAP plays a pivotal role in basal cell differentiation. Specifically, within the trachea, sustained YAP activation inhibits stem cell differentiation while promoting the dedifferentiation of differentiated cells into basal stem cells [23, 40–42]. YAP activation stimulates epidermal keratinocytes proliferation while suppressing differentiation, whereas YAP inhibition reverses these features [43]. Similar results were observed in our work: YAP activation promoted dedifferentiation of differentiated organoids, which warrants further investigation. Unexpectedly, within the intestinal epithelium, YAP overexpression leads to the rapid loss of intestinal crypts, whereas YAP deficiency results in increased proliferation of intestinal stem cells [44]. Conversely, in alveolar, colonic, and ovarian surface epithelium, YAP activation induces epithelial repair after injury [45–47]. There is compelling evidence suggesting that the rumen may have evolved from the esophagus [48, 49]. In the esophagus, the continuous accumulation of YAP promotes basal stem cells proliferation without affecting differentiation [20], which differs from our findings in the rumen, where sustained YAP activation inhibits the differentiation of rumen epithelial basal cells. Thus, it becomes evident that YAP exerts context-specific regulatory effects on epithelial cells.

Tight junctions, gap junctions, adherens junctions, and desmosomes play crucial roles in facilitating adhesion and aggregation among RECs [14, 50, 51]. Among these, tight junctions and adherens junctions form the apical junction complex in polarized epithelial cells, mediating paracellular permeability for ions and solutes and intercellular cell adhesion, respectively [52, 53]. Claudins, a vital family of integral membrane proteins essential for tight junction formation, have been shown to be a key regulator in maintaining the integrity of epithelial barriers, polarity, and overall cell function in various organs [54, 55]. Adherens junctions, on the other hand, are responsible for preserving tissue homeostasis and promoting cell-to-cell communication and signaling [56]. Hippo signaling relies on the neurofibromatosis type 2 (NF2)/Merlin (Mer) and Expanded (Ex) FERM-domain adaptor proteins, which are localized to tight junctions and adherens junctions within epithelial cells [57–59].The involvement of YAP in various cellular junctions has been noted. For instance, YAP has been demonstrated to participate in the formation of junctions between corneal epithelial cells [60], regulate the dynamics of adherens junctions during vascular development [61], influence tight junction integrity within the blood–brain barrier [62], and affect tight junctions in gastric cancer cells [43]. Our research similarly uncovered that YAP activation leads to an increase in the expression of genes associated with tight junctions in organoids. Conversely, YAP inhibition results in a reduction in the expression of genes related to both tight junctions and adherens junctions, affects epithelial barrier integrity, and may potentially explain the disintegration of organoids following Verteporfin treatment. Cell adhesion proteins are strategically located at sites of cell-to-cell contact, making them well-suited for transmitting extracellular signals that regulate the maintenance of normal epithelial function. Further investigations are needed to explore whether YAP affects signal transduction through cell junction.

The process of EMT involves the transformation of epithelial cells into a mesenchymal phenotype, accompanied by the loss of cell polarity and the disruption of cell junctions [63]. YAP has been found to interact directly or indirectly with EMT transcription factors, and extensive studies have indicated a potential link between YAP and EMT [64, 65]. As early as 2006, YAP activation was reported to induce EMT in mammary epithelial cells [66]. Notably, our research suggests that YAP activation or inhibition does not significantly influence the expression levels of EMT-related transcription factors in organoids. This indicates that, within the rumen epithelium, the abnormal accumulation of YAP may not trigger EMT, hinting at the possibility that YAP might impact cell junctions through alternative mechanisms.

In addition to GA-017 and Verteporfin, numerous small-molecule drugs have been reported to possess the capability to modulate the hippo signaling pathway, including TDI-011536, VT02956, MSC-4106, and EMT inhibitor-1 [67–70]. Furthermore, several bioactive compounds extracted from plants have been demonstrated to influence the hippo signaling pathway. For instance, shikonin prevents myocardial damage and induces apoptosis of human T lymphoma cells by activating hippo signaling pathway [71, 72]. Resveratrol has been widely recognized for its regulatory effects on the hippo signaling pathway. It induces YAP nuclear translocation to alleviate hypoxia-induced cardiomyocyte apoptosis [73], inhibits YAP to suppress gastric cancer cell migration, invasion, and growth [74], inhibits YAP activity to counteract hepatic cell fibrosis [75], and activates the hippo signaling pathway to inhibit tumorigenesis of follicular thyroid cancer [76]. Resveratrol has been proven to serve as a nutritional supplement for ruminants, capable of regulating rumen fermentation, methane emissions, and microbial composition [77]. Clearly, the search for suitable exogenous compounds to regulate the hippo signaling pathway in rumen epithelium and assess their impact on animal growth and production is an intriguing avenue of research.

## 5. Conclusions

In this study, we discovered the involvement of the hippo signaling pathway in regulating the morphogenesis of rumen epithelial organoids in vitro. By administering Verteporfin and GA-017, which respectively inhibit and activate YAP, we demonstrated the pivotal role of YAP in the proliferation and differentiation of rumen epithelial basal cells. Our findings provide important insights into the mechanism underlying rumen epithelium development and offer compelling evidence that YAP is a key regulator of rumen epithelial proliferation.

## Figures and Tables

**Table 1:** List of reagents to prepare OCM.

OCM	Final	Source
Advanced DMEM/F-12	—	Gibco
Penicillin/streptomycin	1×	Solarbio
N2	1×	Gibco
B27	1×	Gibco
GlutaMax	1×	Gibco
HEPES	1×	Solarbio
Nicotinamide	10 mM	Sigma
N-acetyl-L-cysteine	1 mM	Sigma
EGF	50 ng/mL	MCE
Noggin	100 ng/mL	MCE
Wnt3a	100 ng/mL	MCE
R-spondin1	100 ng/mL	MCE
IGF-1	100 ng/mL	MCE
FGF-10	100 ng/mL	MCE
CHIR-99021	3 μM	MCE
A83-01	5 μM	MCE
SB202190	10 μM	MCE
Y-27632	10 μM	MCE

**Table 2:** List of antibodies used for immunofluorescence staining.

Antibodies	Source	Identifier
CD49f rabbit pAb	ABclonal	A3236
IVL rabbit pAb	ABclonal	A8026
KRT 10 rabbit mAb	ABclonal	A4669
ZO-1 rabbit pAb	ABclonal	A0659
Ki67 rabbit pAb	Abclonal	A11390
YAP1 rabbit pAb	Abclonal	A1002
CDH1 rabbit pAb	Abclonal	A11492
Cy3 goat anti-rabbit IgG (H + L)	Abclonal	AS007
FITC goat anti-rabbit IgG (H + L)	Abclonal	AS011
DAPI	Beyotime	C1002
Hoechst 33258	Beyotime	C1017

**Table 3:** List of primer sequences.

Genes	Forward primers	Reverse primers
*LATS1*	CTTGGATACCACAGCCCGTT	TGGTGTAGCAGATGCTTGGG
*LATS2*	GCTCCCCTTTGCTAACGAGT	GAACGATCTGCTCCTTGCCT
*MOB1A*	GCACTGAAGCAAGCTGTCCA	AGGTGCCAACTCACGCCTAT
*MOB1B*	GCTTCTTGTTTGGGAGTCGC	CCAACTGGTCCTGAACCCAA
*YAP1*	GAGATCCCTGACGATGTGCC	TCATGGCAAAACGAGGGTCA
*TAZ*	TCGCCTGATCGCTGAATGTC	CGCATCTCCACTGCTGACTT
*TEAD1*	CAGTCACCTGCTCCACCAAA	CCCCTGCATGGTGAGGTTTA
*TEAD2*	GAAGTCTCCACCAGTGAGCG	GGGTTGTCTCACTCCTGTCC
*TEAD3*	GGACATCAAGCCCTTTGCAC	GGGGCCCTTTCTCATAGAGC
*TEAD4*	CGTCCCACGATGTGAAACCT	TGGAGGGTCCACGTTCAAAG

## Data Availability

The data that support the findings of this study are openly available in the NCBI Sequence Read Archive at https://www.ncbi.nlm.nih.gov/sra, reference number PRJNA1069966.

## Supporting Information

Supporting Information Figure S1: KEGG enrichment analysis of each cluster. Figure S2: Effects of Verteporfin treatment on the organoid. Figure S3: DEGs analysis and KEGG enrichment. Figure S4: Gene set enrichment analysis between control and GA-017 groups. Figure S5: Gene set enrichment analysis between control and Verteporfin groups.
