# Supplementary material for: Identifying the Role of YAP in the Development of Rumen Epithelium Using 3D Organoid
Source: Stem Cells Int. 2025 Jul 11;2025:5105796. doi: 10.1155/sci/5105796 (PMC12274096; doi:10.1155/sci/5105796)
Supplement: Supporting Information — Figure S1: KEGG enrichment analysis of each cluster. Figure S2: Effects of Verteporfin treatment on the organoid. Figure S3: DEGs analysis and KEGG enrichment. Figure S4: Gene set enrichment analysis between control and GA-017 groups. Figure S5: Gene set enrichment analysis between control and Verteporfin groups. [file 5105796.f1.docx]

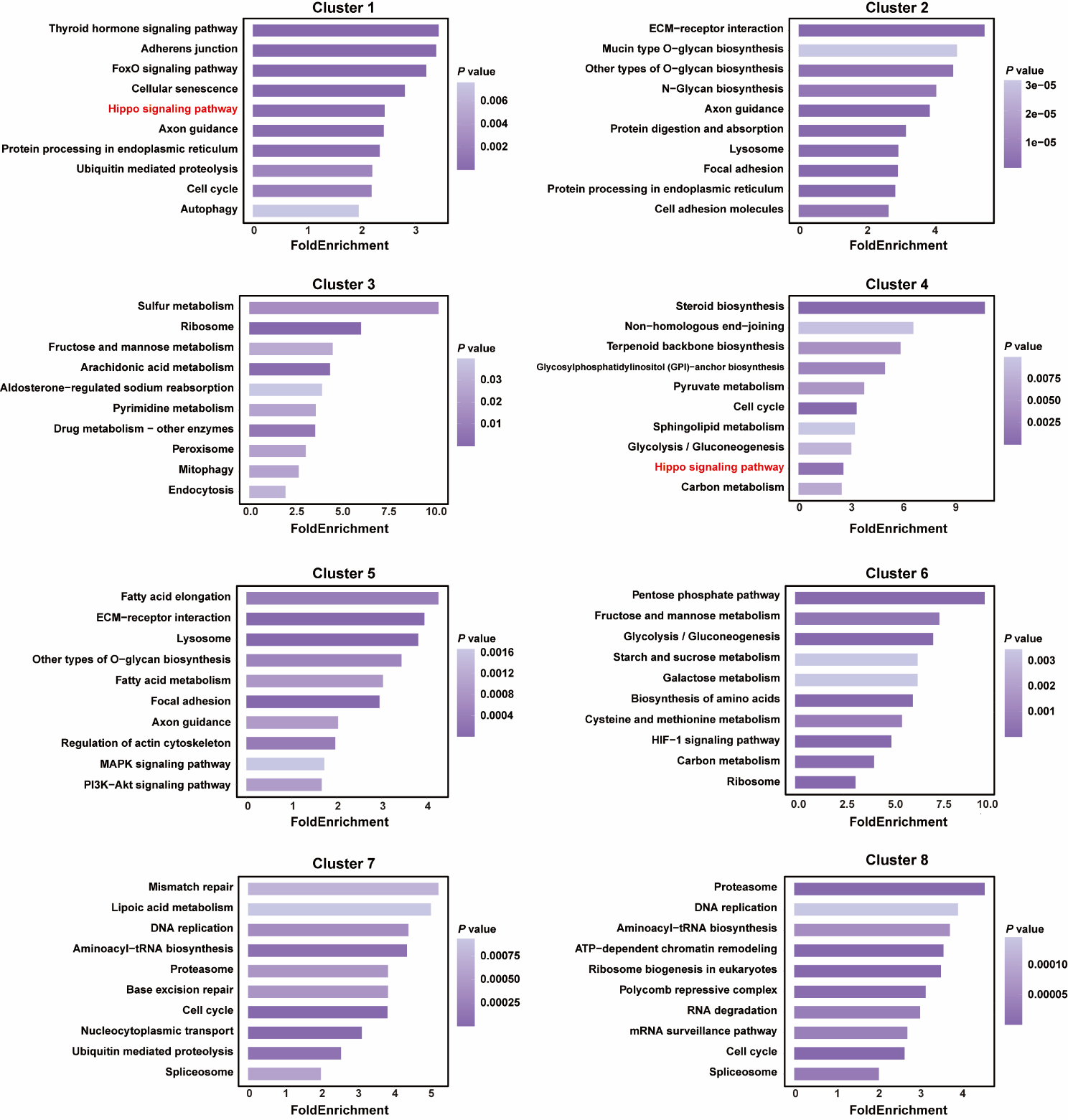


**Fig. S1. KEGG enrichment analysis of each cluster.**


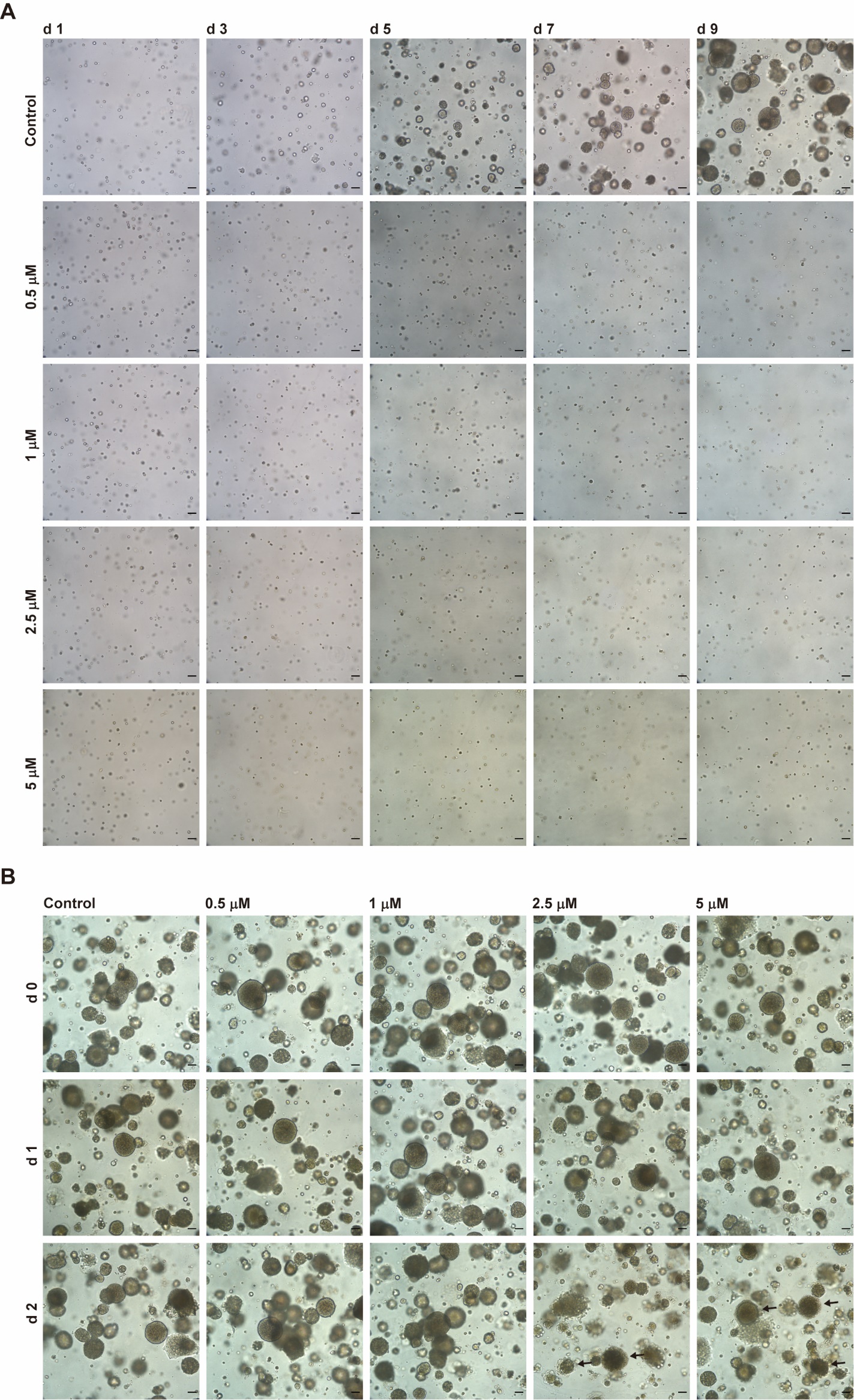


**Fig. S2. Effects of Verteporfin treatment on the organoid. A.** Representative images of organoids treated with different concentration of Verteporfin. **B.** Representative images of mature organoids treated with different concentration of Verteporfin for 2d. Scale bars = 100 μm.


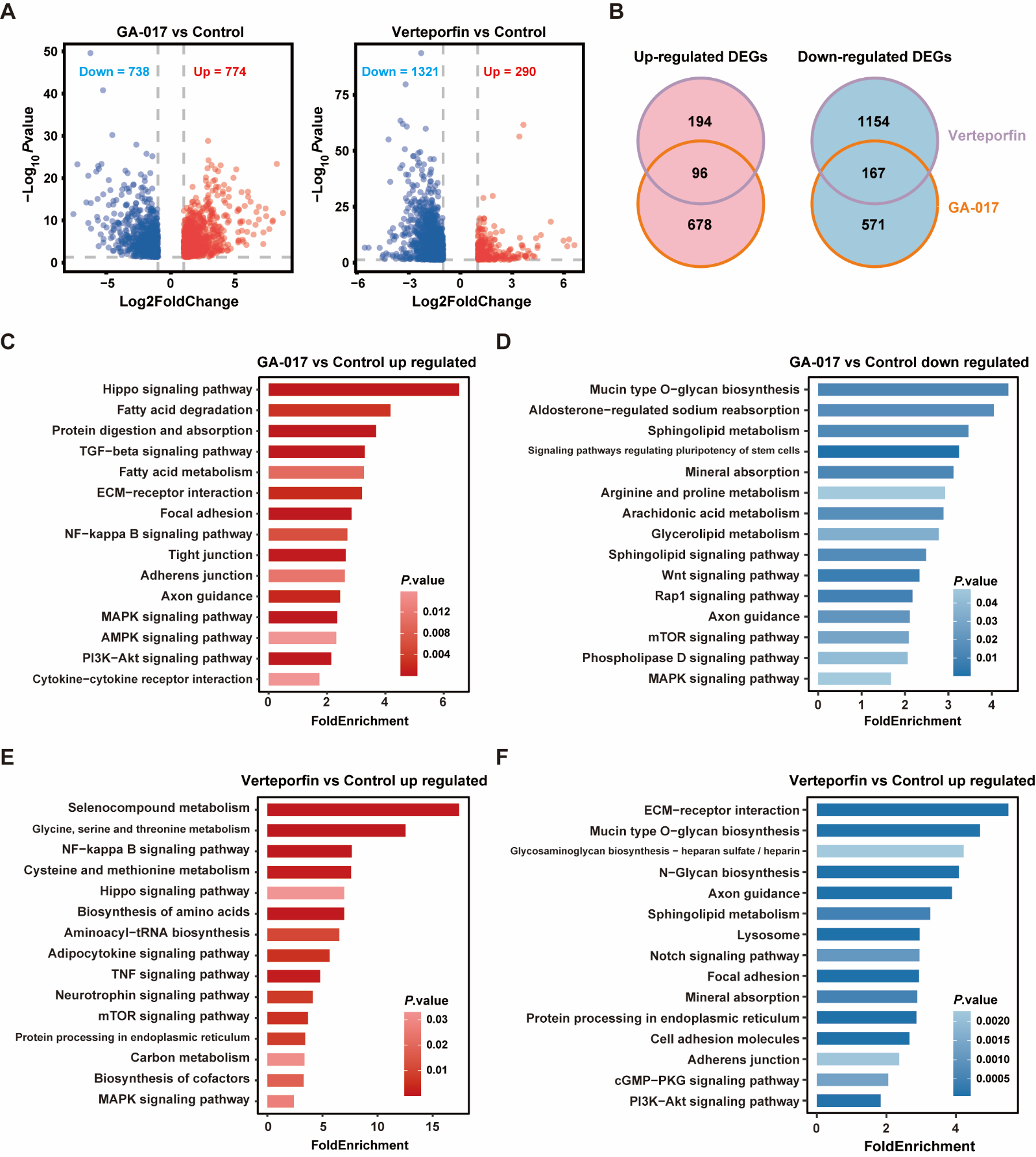


**Fig. S3. DEGs analysis and KEGG enrichment. A.** The DEGs between control group and treatment group (GA-017 and Verteporfin group). **B.** The intersection of up/down-regulated DEGs among different treatment groups. **C-D.** KEGG enrichment analysis of DEGs between control group and GA-017 group. **E-F.** KEGG enrichment analysis of DEGs between control group and Verteporfin group.


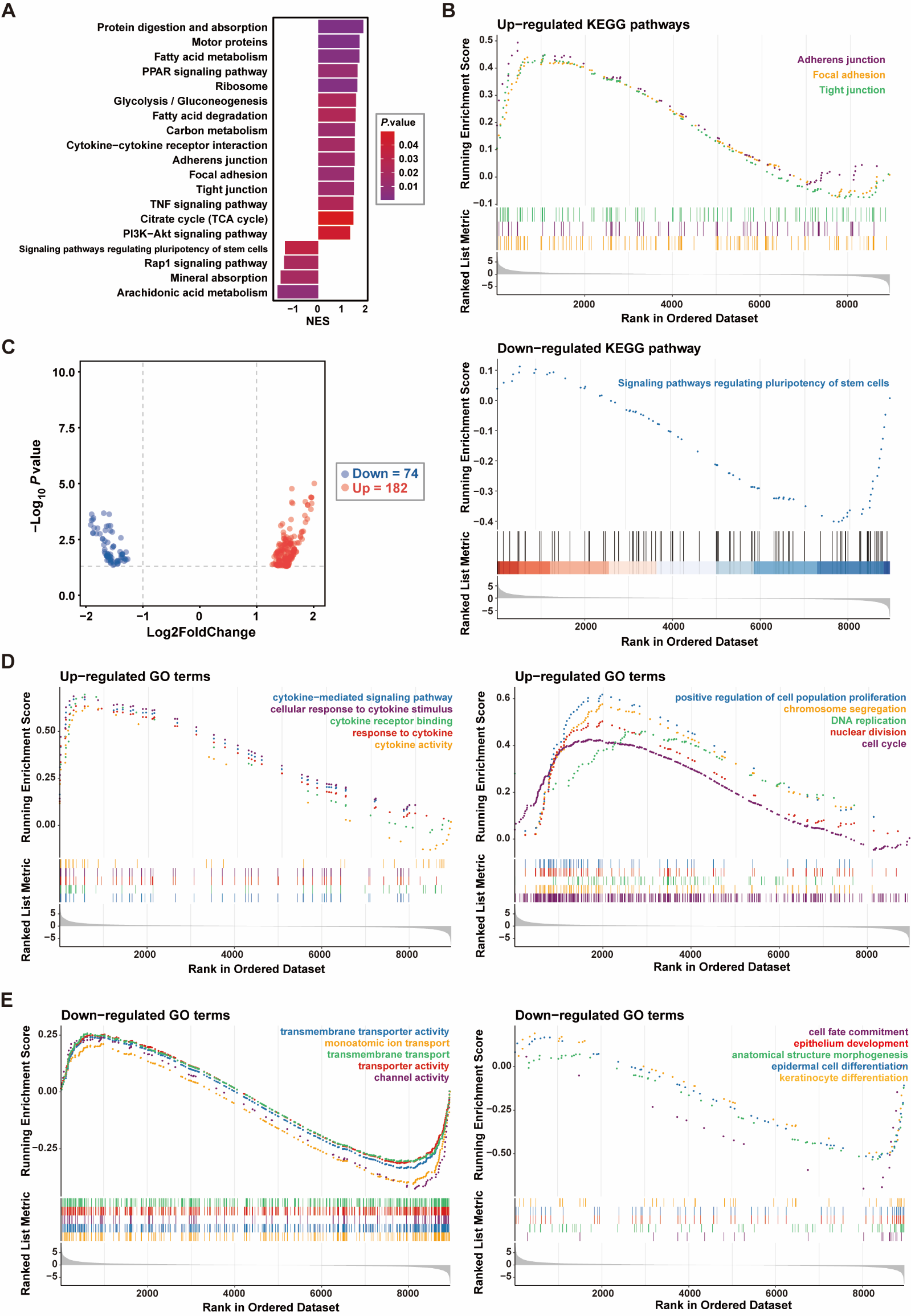


**Fig. S4. Gene set enrichment analysis between control and GA-017 groups. A.** Bar plot shows all KEGG pathways with significant differences between control and GA-017 groups. **B.** Representative KEGG enrichment plots of GSEA between control and GA-017 groups. **C.** Volcano plot shows all GO terms with significant differences between control and GA-017 groups. **D-E.** Representative GO enrichment plots of GSEA between control and GA-017 groups.


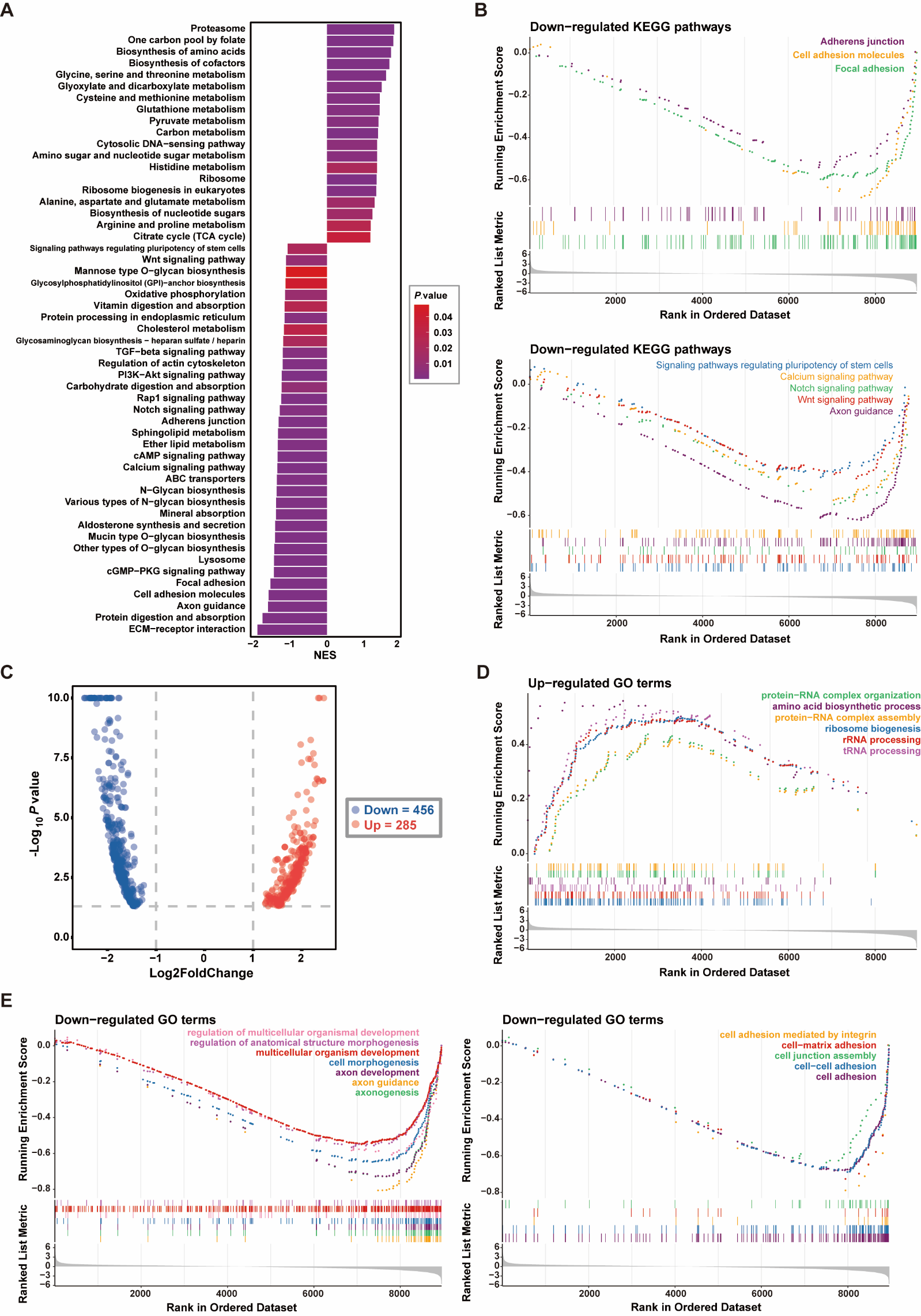


**Fig. S5. Gene set enrichment analysis between control and Verteporfin groups. A.** Bar plot shows all KEGG pathways with significant differences between control and Verteporfin groups. **B.** Representative KEGG enrichment plots of GSEA between control and Verteporfin groups. **C.** Volcano plot shows all GO terms with significant differences between control and Verteporfin groups. **D-E.** Representative GO enrichment plots of GSEA between control and Verteporfin groups.
